# Endoscopic enucleation of the prostate (EEP). The same but different—a systematic review

**DOI:** 10.1007/s00345-021-03705-6

**Published:** 2021-05-06

**Authors:** M. Pallauf, T. Kunit, C. Ramesmayer, S. Deininger, T. R. W. Herrmann, L. Lusuardi

**Affiliations:** 1 Department of Urology, University Hospital Salzburg, Paracelsus Medical University, Salzburg, Austria; 2 Department of Urology, Spital Thurgau AG, Frauenfeld, Switzerland

**Keywords:** EEP, Systematic review, HoLEP, ThuLEP, DiLEP, BipolEP

## Abstract

**Purpose:**

Various techniques for EEP exist. They differ by surgical steps and the source of energy. It is assumed that the latter is of minor importance, whereas adherence to the anatomical enucleation template determines the postoperative result. So far, no systematic review highlights the differences between the energy sources in use for anatomical EEP. This study will address selfsame topic.

**Methods:**

A systematic review of the literature was completed on September 1st, 2020. Studies comparing HoLEP, ThuLEP, DiLEP, or BipolEP with TUR-P providing 12 months of postoperative follow-up were included. Two frequentist network meta-analyses were created to compare the techniques of EEP indirectly.

**Results:**

31 studies, including 4466 patients, were found eligible for our meta-analysis. Indirect pairwise comparison showed differences in surgery time between BipolEP and HolEP (MD − 16.72 min., 95% CI − 27.75 to − 5.69) and DiLEP and HoLEP (MD − 22.41 min., 95% CI − 39.43 to − 5.39). No differences in the amount of resected prostatic tissue, major and minor complications and postoperative catheterization time were found. The odds for blood transfusions were threefold higher for BipolEP than for HoLEP (OR 3.27, 95% CI 1.02–10.5). The difference was not statistically significant when comparing prospective trials and matched-pair analysis only (OR 3.25, 95% CI 0.94–11.18). The Qmax 12 months after surgery was 2 ml/sec. higher for BipolEP than for DiLEP (MD  2.00, 95% CI 0.17–3.84) and 1.94 ml/sec. lower for DiLEP than for HoLEP (MD − 1.94, 95% CI − 3.65 to − 0.22).

**Conclusion:**

The energy source used for EEP has an impact on the intervention itself. BipolEP promotes surgical efficiency; laser techniques lower the risk of bleeding.

**Registry:**

This meta-analysis is registered in the PROSPERO international prospective register registry with the registration number CRD42020205836.

## Introduction

For decades transurethral resection of the prostate (TUR-P) and open prostatectomy (OP) had been the only treatment options for patients with a benign prostatic enlargement (BPE) nonresponding to or unsuitable for pharmacological treatment. However, in large adenomas, greater than 80 ml (ml), there was an unmet need for a different surgical technique, lowering intraoperative blood loss, the risk for complications and comorbidities. The missing link was found with endoscopic enucleation of the prostate (EEP), gaining popularity in treating mid- and small-size glands as well.

Preceding the “EEP era”, there has been an evolution of technical and surgical changes lasting several decades. It all started in 1983 when Y. Hiraoka presented the first EEP technique, where a monopolar detachment probe was used to dissect the prostatic tissue along the surgical capsule to release the prostatic adenoma [1]. Although this technique did not gain any traction, it is considered the blueprint for all EEP techniques. However, the true frontrunner in EEP is HoLEP, introduced by Fraundorfer and Gilling in 1998 [2], the first among the EEP techniques that managed to prove that endoscopic enucleation is as effective as OP while it significantly lowers the surgical morbidity [3, 4]. The “success of HoLEP” was ambiguous. Although the technique attracted interest, it remained one with limited reach due to the lack of teaching opportunities available. Thus, inspired by the concept of minimally invasive surgery, a variety of other endoscopic enucleation methods have emerged. Right from the outset of the “EEP era” in 2006, the group of Fraundorfer and Gilling, who already invented HoLEP, presented the first alternative technique to HoLEP, plasmakinetic i.e. bipolar enucleation of the prostate (PkEP or BipolEP). Over the past years, other bipolar generators have been introduced to the market which no longer follow the principle of plasmakinetic resection. Therefore, the acronym BipolEP is applied in the remainder of the manuscript. The surgical concept of BipolEP and HoLEP is identical. It is not surprising that BipolEP demonstrated comparable results to HoLEP right from the start [5] and therefore advancing to HoLEPs most compelling alternative. Nevertheless, it took an additional 10 years before BipolEP was recognized as a fully valid alternative to HolEP and, together with HolEP, deemed the standard of care for the surgical treatment in BPO-related (benign prostatic obstruction) LUTS in men with prostatic glands greater than 80 ml [6]. These conclusions were based on the results of two meta-analyses comparing BipolEP and HoLEP with OP showing similar surgical efficacy leading to lasting relief of prostatic obstruction in addition to an advantageous perioperative profile of shorter postoperative catheterization and hospital stay and a reduced need for blood transfusions [7, 8]. Built on these positive results of EEP and the feasibility in using alternative energy sources to the holmium:YAG laser, further laser-based enucleation methods were introduced. The use of the thulium:YAG laser for endoscopic enucleation resulted in the development of two different enucleation techniques, the (i) vapo-enucleating approach (ThuVEP), introduced by Bach et al. in 2009 [9], and the (ii) anatomical enucleation of the prostate (ThuLEP), introduced by TRW Herrmann et al. in 2010 [10]. Although the same laser is used, ThuVEP and ThuLEP differ in main surgical steps, which underscore the laser’s different action mechanisms in endoscopic enucleation. In ThuVEP, the continuous-wave laser is used for cutting and coagulating the prostatic tissue at the same time, putting the focus on bloodless surgery. In ThuLEP, on the other hand, the laser is used for incision and subtle coagulation of the tissue only, whereas the detachment/dissection of the adenoma is performed with blunt force. This approach enables ThuLEP to release the same surgical template as HoLEP, resulting in a similar outcome [11, 12]. Consequently, ThuLEP became the prototype for EEP using a continuous-wave laser. The diode laser is another continuous-wave laser used in EEP. Diode laser enucleation of the prostate (DiLEP) was first presented in 2011 by the study groups of Lusuardi et al. and Buisan et al. [13, 14]. Their techniques were similar ones, yet applied a different laser wavelength. As for ThuLEP, also DiLEP was able to show a comparable outcome to HoLEP [15].

Because of the similarity of the findings for HoLEP, BipolEP, ThuLEP, and DiLEP, which are all considered techniques of anatomical enucleation (AEEP), it was concluded that complete release of the surgical template is the main determining factor for the surgical outcome. In contrast, the energy source in use is less important. Hence, scientific discourse changed abruptly and focused on the technique of anatomical enucleation instead. The phrase “enucleation is enucleation is enucleation is enucleation” published by TRW Herrmann in an editorial comment in the World Journal of Urology in 2016 aptly highlights this turning point in the scientific debate [16].

But how to decide on which EEP technique to use? What are the advantages and disadvantages for holmium-, thulium-, diode laser, or bipolar current when performing EEP? Does one fit all, or do we need to decide for every patient on a case-to-case basis?

The manuscript aims to overcome this knowledge gap and take patient counseling in BPE treatment to the next level. We reviewed the current evidence on HoLEP, ThuLEP, DiLEP, and BipolEP in a systematic fashion and performed an analysis of their 12-month outcomes.

## Material and methods

### Literature search

From August 2020 to September 2020, a literature search was performed by the authors T.K. and M.P. through the PubMed/Medline to identify studies investigating the outcomes of HoLEP, ThuLEP, DiLEP, and BipolEP. This examination was performed using the following search terms in different combinations: “prostate”, “transurethral”, “enucleation”, “bipolar”, “BipolEP”, “plasmakinetic”, “PkEP”, “laser”, “holmium”, “HoLEP”, “thulium”, “ThuLEP”, “diode”, “DiLEP”.

The reference lists of the studies found were also used to gain additional relevant literature.

### Study selection

**Fig. 1:**
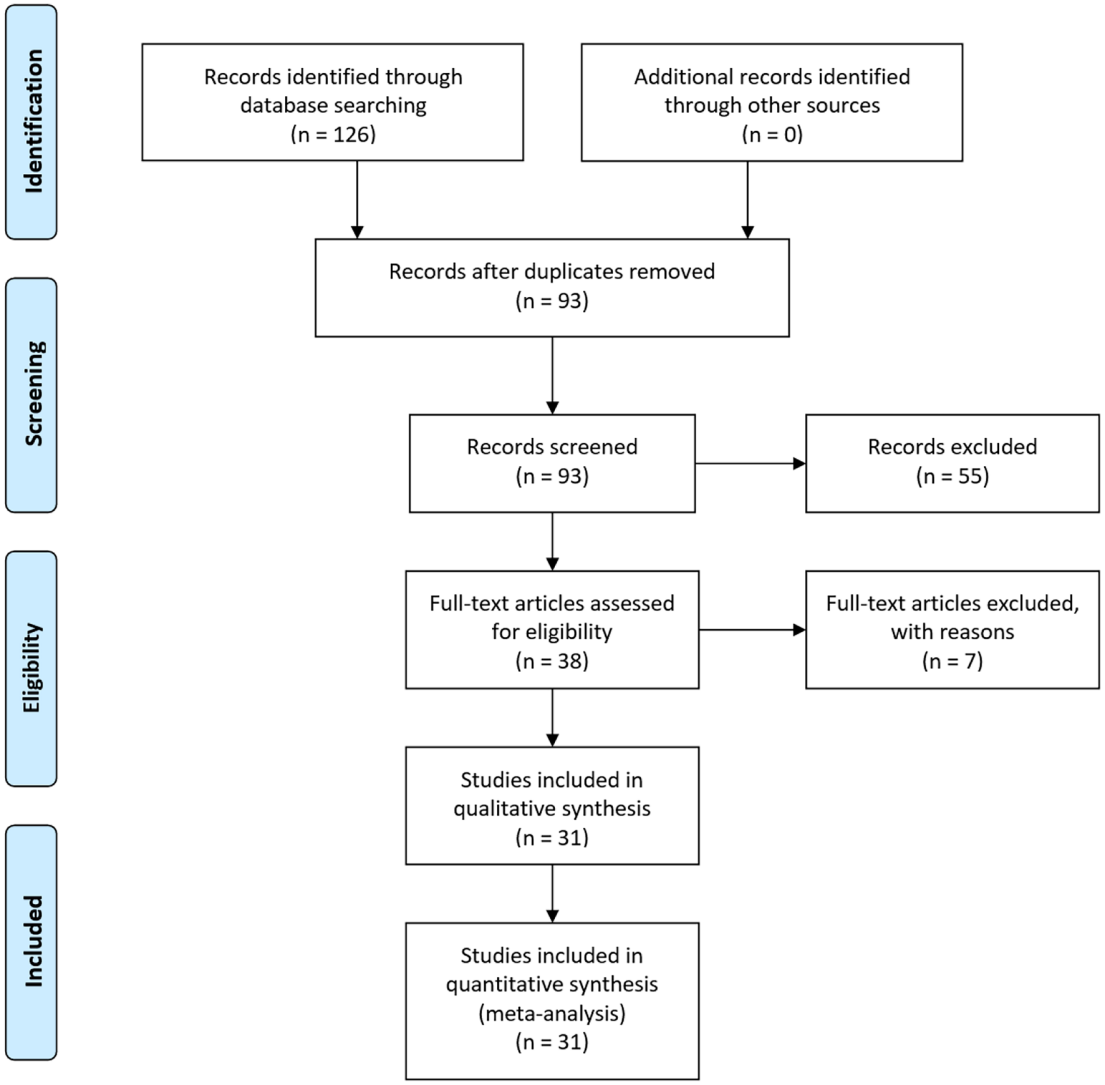
Flow diagram of the study selection process: this graphic provides an overview of the study selection process. The graphic was designed according to the specifications of PRISMA (preferred reporting items for systematic Reviews and Meta-analyses) http://prisma-statement.org/PRISMAStatement/FlowDiagram

Study selection was performed by the authors T.K and M.P. The process followed the recommendations of the PRISMA-Statement (Preferred Reporting Items for Systematic Review and Meta-analysis Statement—www.prisma-statement.org). The flowchart Fig. 1provides an overview of the selection process.

All studies found in the literature search were checked for duplications (including studies reporting the same study population but a different follow-up period). Subsequently, the abstracts were examined for eligibility, and if these criteria were met, the entire publication was reviewed. All comparative studies comparing one of the enucleation methods (HoLEP, ThuLEP, DiLEP, and BipolEP) with the standard procedure TUR-P for the treatment of BPE-related LUTS and providing information on the intervention and a follow-up period of 12 months were included in our analysis. Not all parameters of interest had to be listed in the publication. At least one intra- and one postoperative study endpoint had to be given. Publications were only included if both authors agreed that all requirements had been fulfilled.

### Assessment of study quality

Assessment of study quality was performed by the authors T.K., M.P., and C.R. Studies were evaluated on the following aspects: the study’s level of evidence was assessed according to the recommendations of the “Oxford Level of Evidence Working Group” [17]. The quality of the study was assessed using the “Jadad Scale” [18] for randomized trials and the “Newcastle–Ottawa Assessment Scale” (NOS) [19] for non-randomized comparative trials. The study was classified as low quality with 0–2 points on the “Jadad Scale” or 0–5 points on the NOS, intermediate quality with 3 points on the “Jadad Scale” or 6 points on the NOS, and high quality with 4–5 points on the “Jadad Scale” or 7–9 points on the NOS.

### Data extraction

Data extraction was conducted by the authors T.K., M.P., and C.R. For this purpose, a uniform data table was used, which queried the following information: general information of the publication (name, authors, journal, and year of publication), type of study, baseline characteristics (number of patients, mean value and standard deviation of age, prostate volume, prostate-specific antigen (PSA) level, IPSS, Qmax and PVR, and the absolute number of patients with an indwelling catheter), perioperative information (costs, mean value and standard deviation of the operation time, the weight of the resected prostatic tissue and postoperative catheterization time and all complications rated according to the Clavien–Dindo classification (CD)) as well as follow-up data (mean value and standard deviation of IPSS, PVR, Qmax and quality of life (QoL) at 1,3,6, and 12 months after surgery, difference in prostate-specific antigen (PSA) level before and 12 months after surgery, sexual function (International Index of Erectile Function Questionnaire (IIEF)) and incontinence rate 12 months after surgery). If the complications listed in the publication had not been classified according to the CD, the investigators reclassified them independently. For this purpose, it was assumed that the measures that were taken to treat the complications complied with the recommendations of the European Association of Urology (EAU) Guidelines.

### Statistical analysis

Two frequentist network meta-analyses were created to directly compare the reference operation method TUR-P against all other operation methods and to compare the latter methods indirectly. All direct and indirect pairwise comparisons between all operations methods are presented using appropriate effect sizes (for continuous variables, the mean differences (MD), and for dichotomous variables, odds ratios (OR) were used) and two-sided 95% confidence intervals (CI). Based on the estimated heterogeneity between the studies, which was assessed using Higgins *I*^2^ and Cochran’s Q, a fixed-effects or random-effects model was used. Funnel plots and Egger’s test for asymmetry were used to assess potential publication bias.

A *p* value < 0.05 was taken as the uncorrected statistical significance level (two-sided). Therefore, all inferential results are only descriptive. For statistical analysis, the statistical computing software R Version 4.0.2 (R Foundation for Statistical Computing, Vienna, Austria. URL http://www.R-project.org) was used. For conducting the network meta-analysis, the R package netmeta *(Gerta Rücker, Ulrike Krahn, Jochem König, Orestis Efthimiou and Guido Schwarzer (2020). netmeta: Network Meta-Analysis using Frequentist Methods. R package version 1.2–1.)* was used.

## Results

### Study characteristics

#### Study selection

In total, 31 studies were found eligible for our analysis—21 prospective randomized trials, of which 11 compare HoLEP [20–30], 3 ThuLEP [31–33], 2 DiLEP [13, 34], and 5 BipolEP [35–39] against TUR-P; 2 prospective cohort studies [40, 41], 1 matched-pair analysis [42], and 6 retrospective cohort studies [43–48] comparing BipolEP against TUR-P; 1 retrospective cohort study [49] comparing DiLEP against TUR-P. In total, data of 4466 patients were enrolled in our analysis, of which 2201 had been treated with TUR-P, 672 with HoLEP, 235 with ThuLEP, 180 with DiLEP, and 1178 with BipolEP.

The primary meta-analysis included the results of all 31 publications. All endpoints were analyzed. The second meta-analysis included the results of all prospective trials as well as the matched-pair analysis. The endpoints “duration of postoperative catheterization”, “perioperative blood transfusions”, “complications Clavien-Dindo I-IIIA” and “complications Clavien-Dindo IIIB-V” were analyzed.

#### Level of evidence and study quality

The assessment of the selected studies for their level of evidence (LoE) revealed 5 studies with LoE 1 (16.13%), 18 studies with LoE 2 (58.06%), 1 study with LoE 3 (3.23%), 7 studies with LoE 4 (22.58%) and no study with LoE 5 (0%).

Of the randomized studies, 11 studies (52.34%) were rated low quality (“Jadad Scale” 0–2 points), 8 (28.91%) intermediate quality (“Jadad Scale” 3 points), and 2 (9.52%) high quality (“Jadad Scale” 4–5 points).

Of the non-randomized studies, no study (0%) was rated low quality (NOS 0–5 points), 1 (10%) intermediate quality (NOS 6 points), and 9 (90%) high quality (NOS 7–9).

#### The risk for publication bias

Using funnel plots and Egger’s test for asymmetry, a possible publication bias for the outcomes “resected tissue weight” (*p* = 0.0003), IPSS score at 6 months (*p* = 0.0029) and 12 months (*p* = 0.0252) were found. The Funnel plots showed no distortion for the remaining results, and the Egger’s test did not show any statistically significant finding, suggesting that a publication bias is unlikely. Even though the funnel plots and the Egger’s test showed indication of a potential publication bias for 3 out of 14 outcomes, the authors selected all relevant available studies for this network meta-analysis to their best knowledge.

#### Baseline characteristics patients

Baseline characteristics for all groups (HoLEP, ThuLEP, DiLEP, and BipolEP) are stated in Table 1

**Table 1:** Preoperative baseline characteristics

		TUR-P	HoLEP	ThuLEP	DiLEP	BipolEP
Age						
*n*_total_		1784	620	235	180	808
* n*_average_		69	62	78	60	81
# of studies		26	10	3	3	10
mean_weighted_		69.15	68.35	68.14	71.27	69.81
SD_weighted_		8.20	7.31	12.73	7.80	7.12
Prostate volume (milliliter)						
*n*_total_		1832	672	235	180	808
* n*_average_		68	61	78	60	81
# of studies		27	11	3	3	10
mean_weighted_		72.94	61.87	77.53	62.42	86.39
SD_weighted_		22.47	24.15	34.13	20.74	20.62
PSA (ng/ml)						
*n*_total_		1109	491	235	30	327
*n*_average_		58	61	78	30	47
# of studies		19	8	3	1	7
Mean_weighted_		4.41	3.21	2.99	3.50	7.19
SD_weighted_		3.32	2.12	3.23	1.22	4.31
IPSS score prior to surgery						
*n*_total_		1811	563	235	106	882
*n*_weighted_		70	56	78	53	98
# of studies		26	10	3	2	9
Mean_weighted_		22.83	23.76	20.86	25.18	23.18
SD_weighted_		4.65	4.08	5.81	3.92	4.78
Qmax prior to surgery						
*n*_total_		1898	663	218	106	923
n_average_		70	60	73	53	92
# of studies		27	11	3	2	10
Mean_weighted_		7.00	6.75	7.97	6.01	6.64
SD_weighted_		3.22	2.68	4.27	2.27	2.49
Post-void residual volume (milliliter)						
*n*_total_		1331	290	218	106	783
*n*_average_		67	48	73	53	87
# of studies		20	6	3	2	9
Mean_weighted_		128.70	133.00	113.16	196.65	137.06
SD_weighted_		83.54	97.68	71.14	167.10	51.77
Percentage of patients with an indwelling catheter prior to surgery						
*n*_total_		1019	342	235	106	405
*n*_average_		68	49	78	53	135
# of studies		15	7	3	2	3
%_weighted_		14.92%	30.12%	7.23%	0.00%	11.60%

This table gives an overview of the patient’s baseline characteristics for all studies included. Only studies with complete information (*n*, MW, SD) were used. Therefore, for each surgical method, the total number of patients (n_total_) can differ between the variables. For the calculation of the aggregated weighted indicators (mean_weighted_, SD_weighted_, %_weighted_) for each surgical method, the weighting was based on the number of cases per study. (*n*_*total*_ total number of cases per surgical method; *n*_*average*_ average number of cases per surgical method, rounded to decimals; *# of studies* number of studies per surgical method)

### Perioperative results

#### Surgery time (including time for removal of the prostatic tissue)

In direct comparison against TUR-P, only HoLEP showed a statistical difference for operation time, yielding a longer mean operation time for HoLEP (MD 15.78 min, 95% CI 8.03–23.53). Regarding all indirect pairwise comparisons between the enucleation methods (HoLEP, ThuLEP, DiLEP and BipolEP), a statistical relevant difference was found in BipolEP vs. HoLEP (MD − 16.72 min, 95% CI − 27.75 to − 5.69) and DiLEP vs. HoLEP (MD − 22.41 min, 95% CI − 39.43 to − 5.39) showing shorter mean operation time for BipolEP and DiLEP.

#### Resected tissue

In direct comparison against TUR-P, only BipolEP showed a statistically relevant difference in the amount of resected prostatic tissue (*p* = 0.001). Mean resected prostatic tissue for BipolEP was 13.65 g higher than for TUR-P (MD 13.65 g, 95% CI 5.43–21.87). Indirect pairwise comparison between all enucleation methods (HoLEP, ThuLEP, DiLEP, and BipolEP) showed no statistically relevant difference.

#### Duration of postoperative catheterization

In direct comparison against TUR-P all endourological enucleation methods (HoLEP, ThuLEP, DiLEP and BipolEP) demonstrated a shorter mean postoperative catheterization time. The difference ranged between 0.45 days for BipolEP (MD − 0.45 days, 95% CI − 0.79 to − 0.11, *p* = 0.009), 0.79 days for HoLEP (MD − 0.79, 95% CI − 1.12 to − 0.47, *p*≦0.001), 1.3 days for ThuLEP (MD − 1.3, 95% CI – 1.9 to − 0.69, *p*≦0.001) and 1.6 days for DiLEP (MD − 1.6, 95% CI − 2.15 to – 1.05, *p*≦0.001). However, indirect pairwise comparison between the enucleation methods showed no statistically relevant difference.

The results of the primary and the second meta-analysis did not differ.

#### Complications

In direct comparison against TUR-P for complications CD I-IIIA (*minor complications),* only HoLEP showed a statistically relevant difference (*p* = 0.023). The odds for complications CD I-IIIA were 52% lower for HoLEP than for TUR-P (OR 0.48, 95% CI 0.25–0.9). Indirect pairwise comparison between the enucleation methods (HoLEP, ThuLEP, DiLEP, and BipolEP) showed no statistically relevant difference.

In direct comparison against TUR-P for complications CD IIIB-V (*major complications),* only BipolEP showed a statistically relevant difference (*p* = 0.015). The odds for complications CD IIIB-V were 46% lower for BipolEP than for TUR-P (OR 0.54, 95% CI 0.32–0.86). Like for complications CD I-IIIA, no statistically relevant differences were found in the indirect pairwise comparison between the enucleation methods (HoLEP, ThuLEP, DiLEP, and BipolEP).

Neither for complications CD I-IIIA nor complications CD IIIB-V, the results differed between the primary and the second meta-analysis.

Direct comparison against TUR-P for perioperative blood transfusions showed statistically relevant differences for HoLEP only (*p* = 0.001). The odds for perioperative blood transfusions were 81% lower for HoLEP than for TUR-P (OR 0.19, 95% CI 0.07–0.49). Indirect pairwise comparison between the enucleation methods (HoLEP, ThuLEP, DiLEP, and BipolEP) showed a statistically relevant difference between BipolEP and HoLEP (OR 3.27, 95% CI 1.02–10.5). The odds for blood transfusions were more than threefold higher for BipolEP than for HoLEP. This was the result of the primary meta-analysis. The second meta-analysis showed no statistically significant difference in the indirect pairwise comparison of BipolEP and HolEP (OR 3.25, 95% CI 0.94–11.18).

#### Costs

None of the selected studies listed the costs for surgery in the publication.

**Fig. 2:**
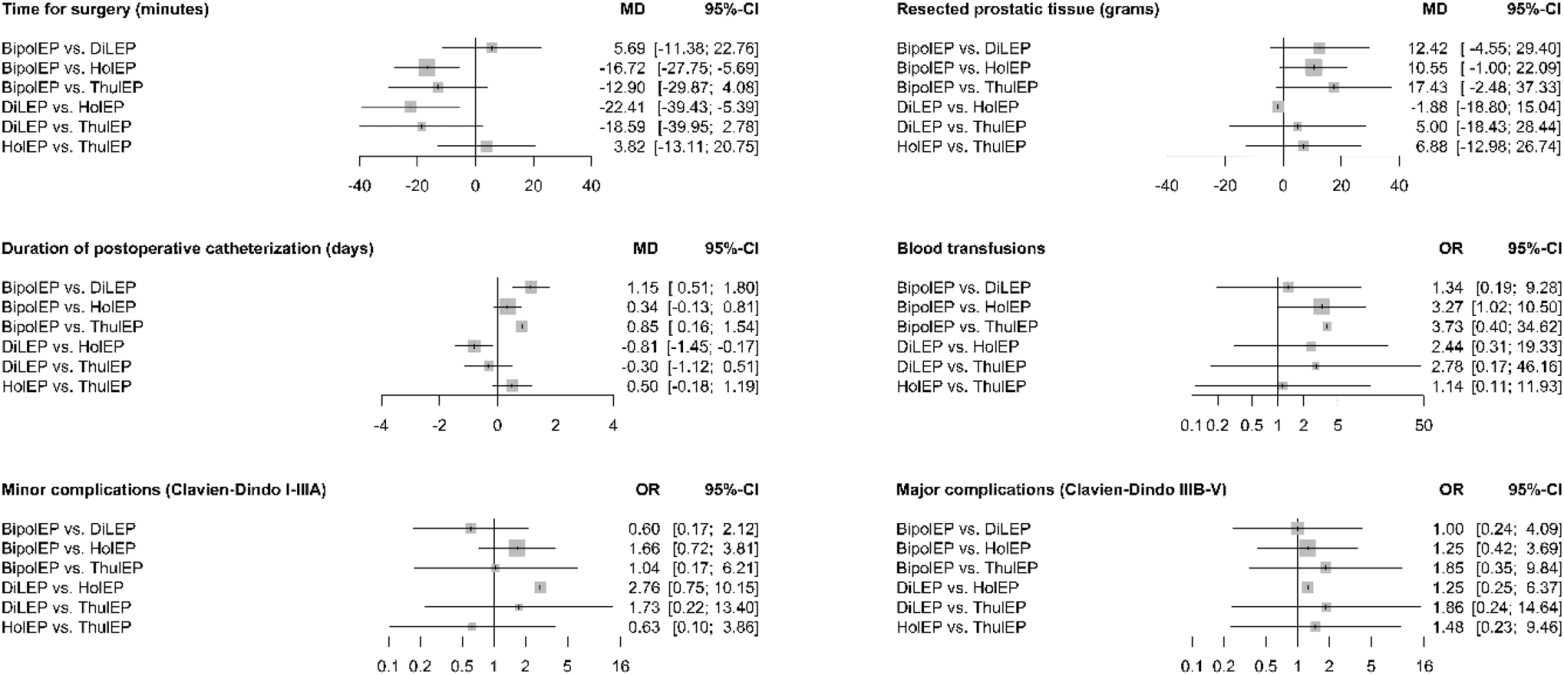
Perioperative results—Meta-analysis one: this graphic shows the results of the indirect pairwise comparison between all enucleation methods. The results are presented using forest plots. For continuous variables, the mean differences, and for dichotomous variables, the odds ratios are given as well as two-sided 95% confidence intervals. (Meta-analysis one includes all 31 studies)

**Fig. 3:**
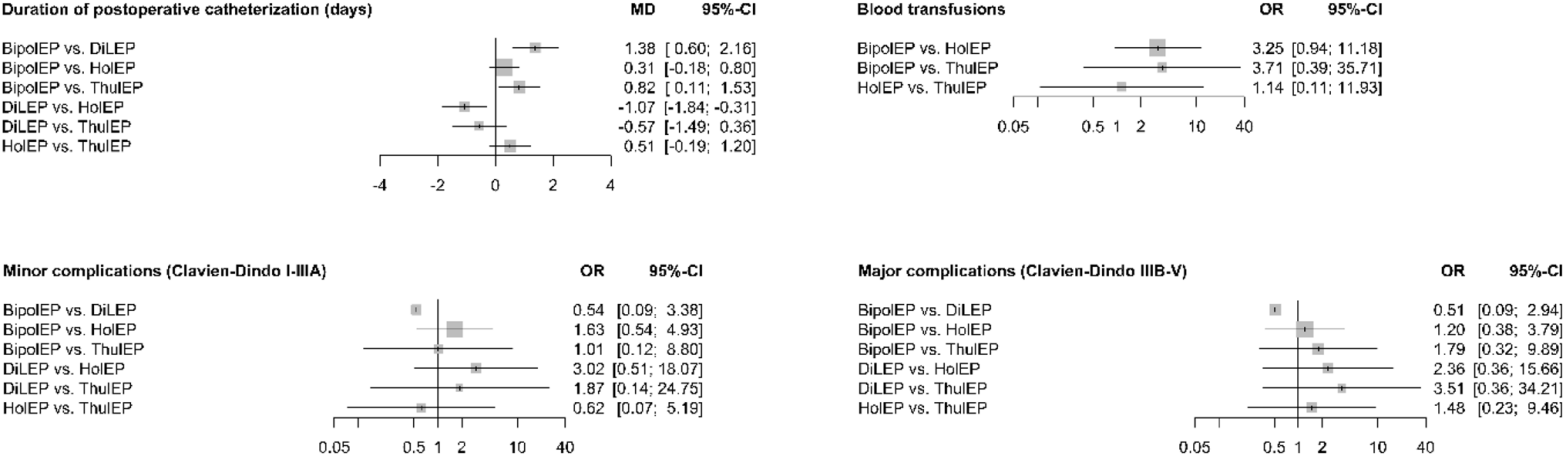
Perioperative results—Meta-analysis two: this graphic shows the results of the indirect pairwise comparison between all enucleation methods. The results are presented using forest plots. For continuous variables, the mean differences and for dichotomous variables, the odds ratios are given as well as two-sided 95% confidence intervals. (Meta-analysis two includes prospective trials and matched-pair analysis only)

An overview of indirect pairwise comparison between all enucleation methods is given in Fig. 2 and Fig. 3

### Functional outcome

#### IPSS and Qmax

No statistically relevant difference was found in direct comparison against TUR-P and indirect pairwise comparison between the enucleation methods (HoLEP, ThuLEP, DiLEP, and BipolEP) for IPSS 1, 3, and 6 months postoperative. Direct comparison against TUR-P showed a statistically significant difference of the IPSS 12 months postoperative for HoLEP (p≦0.001). The mean IPSS of HoLEP was 0.89 points lower than that of TUR-P (MD − 0.89, 95% CI − 1.35 to − 0.44). Indirect pairwise comparison between the enucleation methods (HoLEP, ThuLEP, DiLEP, and BipolEP) showed a statistically significant difference of the IPSS between HoLEP and ThuLEP 12 months after surgery. The mean IPSS of HoLEP was 1.49 points lower than that of ThuLEP (MD − 1.49, 95% CI − 2.9 to − 0.08).

No statistically relevant difference was found in direct comparison against TUR-P for Qmax at postoperative months 1 and 6 and indirect pairwise comparison between the enucleation methods (HoLEP, ThuLEP, DiLEP, and BipolEP) for Qmax at postoperative month 1, 3, and 6. Three months after surgery, direct comparison against TUR-P for Qmax showed a statistically significant difference for HoLEP (*p* = 0.042). The mean value of Qmax was 2.41 ml/sec. higher for HoLEP than for TUR-P (MD 2.41, 95% CI 0.09–4.74). Twelve months after surgery, direct comparison against TUR-P for Qmax showed statistically significant differences for BipolEP (*p* = 0.014) and HoLEP (*p*≦0.001). The mean value of Qmax was 1.1 ml/sec. higher for BipolEP (MD 1.1, 95% CI 0.23–1.98) and 1.04 ml/sec. higher for HoLEP (MD 1.04, 95% CI 0.46–1.61). Indirect pairwise comparison between the enucleation methods (HoLEP, ThuLEP, DiLEP, and BipolEP) for Qmax 12 months after surgery showed statistically significant differences for BipolEP vs. DiLEP and DiLEP vs. HoLEP. BipolEPs and HoLEPs mean value of Qmax were 2 ml/sec. (MD 2.00, 95% CI 0.17–3.84) and 1.94 ml/sec. (MD − 1.94, 95% CI − 3.65 to − 0.22) higher than DiLEPs.

#### The difference in PSA level before and 12 months after surgery

Only 8 out of 31 publications presented data on PSA-value differences before and 12 months after surgery. No publication on ThuLEP or DiLEP, one publication on HoLEP, and seven publications on BipolEP presented data for this endpoint. Due to a lack of data on PSA-value differences, no statistical analysis was performed.

#### Incontinence rate 12 months after surgery

Sixteen out of 31 publications presented data on incontinence rates 12 months after surgery. No publication on ThuLEP and only one publication on DiLEP presented data for this endpoint. The latter with no event of incontinence in both study groups. Eighth publications on BipolEP presented data for this endpoint. However, there was no incontinence event in the BipolEP group and only one in the TUR-P group. Seven publications on HoLEP presented data for this endpoint. Incontinence occurred in both groups. Because of the lack of data for ThuLEP, DiLEP, and BipolEP, no statistical analysis was performed.

#### International index of erectile function questionnaire 12 months after surgery

Only 5 out of 31 publications presented data on postoperative sexual function, i.e., on the IIEF questionnaire. No publication on ThuLEP and DiLEP, one publication on BipolEP, and four publications on HoLEP presented data for this endpoint. Due to a lack of data on postoperative sexual function, no statistical analysis was performed.

**Fig. 4:**
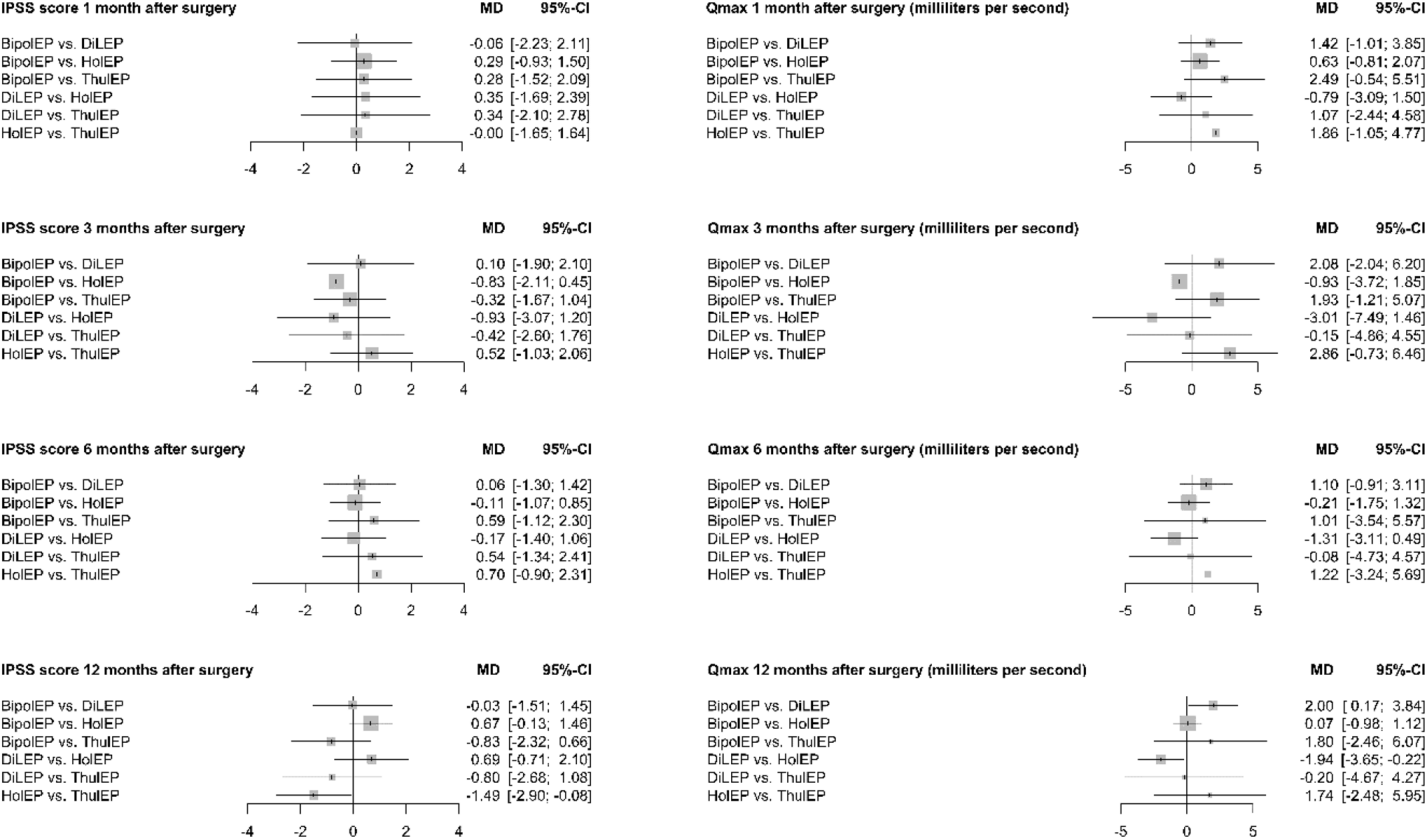
Functional outcome—Meta-analysis one: this graphic shows the results of the indirect pairwise comparison between all enucleation methods. The results are presented using forest plots. For continuous variables, the mean differences and for dichotomous variables, the odds ratios are given as well as two-sided 95% confidence intervals. (Meta-analysis one includes all 31 studies)

An overview of indirect pairwise comparison between all enucleation methods is given in Fig. 4.

## Discussion

If we compare the number of prospectively randomized trials included in this meta-analysis between the enucleation methods (HoLEP, ThuLEP, DiLEP, and BipolEP), a significant imbalance emerges. Overall, there are 11 prospective randomized trials for HoLEP, 5 for BipolEP, 3 for ThuLEP, and only 2 for DiLEP. If we also compare the number of patients included in this meta-analysis for each surgical technique (TUR-P, HoLEP, ThuLEP, DiLEP, and BipolEP), the discrepancy becomes even more lucid. Two-thousand-two-hundred-one patients had been treated with TUR-P, 1178 with BipolEP, and 672 with HoLEP. In contrast, only 235 patients had been treated with ThuLEP and 180 with DiLEP. This inequality of available data cannot be ignored in the interpretation of this study's findings. It might explain why there is hardly any difference in the results between the laser-based enucleation methods. Consequently, this meta-analysis only allows drawing proper conclusions on the comparison between enucleation and TUR-P and enucleation using a laser system and enucleation using electric current. Even if the resectoscope's energy source is of minor importance, the different physical properties of lasers and electric current in treating the prostatic tissue cannot be dismissed. Being aware of them proves key to good patient counseling. This meta-analysis is intended to simplify this challenging task.

Endoscopic enucleation and TUR-P differ significantly in postoperative catheterization duration, which is shorter with all enucleation techniques—leading to the conclusion that endoscopic enucleation reduces postoperative haematuria. One possible explanation is that during enucleation, the prostatic tissue is being dissected directly beneath the capsule. The blood vessels are only cut and coagulated once, promoting bloodless surgery. In contrast, in TUR-P, the vessels are cut open each time the prostatic tissue is being resected. However, such conclusions cannot be drawn solely based on differences in postoperative catheterization duration, which does not depend on the postoperative course of haematuria only, but also on the treating physicians’ preferences and common practice. It is at high risk of being biased.

Information on postoperatively administered blood transfusions provides more reliable data for interpreting interoperative hemorrhage. However, it only provides information about the risk of severe bleeding events and not bleeding in general. Data show that using a laser reduces the risk for severe hemorrhage. In our meta-analysis, the odds for postoperative blood transfusions were 81% lower for HoLEP than TUR-P and threefold higher for BipolEP than HoLEP. This finding is supported by numerous studies that demonstrate excellent hemostasis when enucleating prostatic tissue with a laser [5, 11, 15]. The lasers’ excellent coagulation properties result from the ability to penetrate the tissue below the resection line’s surface and cause hemostasis [50]. Evaluating the rate of postoperative blood transfusions also shows that the surgical technique (EEP vs. TUR-P) impacts the bleeding risk. Because in the second meta-analysis, including all prospective trials and matched-pair analysis, there was no statistically significant difference between HoLEP and BipolEP anymore, whereas the difference between HoLEP and TUR-P persisted.

We conclude, both the energy source and the surgical technique influence intraoperative hemorrhage, whereas the latter is most important.

In comparison with TUR-P, enucleation techniques harbor a lower complication risk. In direct comparison with TUR-P, the odds for minor complications (CD I-IIIA) were 52% lower for HoLEP and the odds for major complications (CD IIIB-V) 46% lower for BipolEP. However, indirect pairwise comparison between the enucleation methods (HoLEP, ThuLEP, DiLEP, and BipolEP) did not show any statistical difference. These results further strengthen the safety profile of endoscopic enucleation. However, not in all studies under investigation, complications had been assessed according to the CD, and therefore this had to be carried out retrospectively by our study team. This evidently reduces the reliability of our findings. On the other hand, the second meta-analysis, including prospective trials and matched-pair analysis only, showed similar results, which again supports our study’s validity.

Two major complications, the impact on sexual function—measured by the difference in the IIEF score, and the rate of postoperative urinary incontinence (UI), were not adequately reported in the selected studies. That these endpoints are missing in our meta-analysis weakens this study’s message. In terms of postoperative UI, a distinction has to be made between the two forms of postoperative UI, “immediate” urge urinary incontinence (UUI) and “prolonged” stress urinary incontinence (SUI). The former is highly relevant for EEP as it depends on the surgeons’ surgical experience. A prospective study on the learning curve of HoLEP showed a UI rate of 28.5% one month after surgery, dropping to 8.6% and 2.2% 4 and 12 months postoperatively. Surgeons who had performed more than 20 procedures had a lower UI rate at 1 and 4 months compared to less experienced colleagues. Overall, spontaneous remission of early UI was high, resulting in a low rate of long-term UI. [51] Low rates for SUI had been confirmed by a meta-analysis comparing the EEP techniques HoLEP, ThuLEP, and BipolEP with TUR-P, showing no difference between the EEP group and TUR-P. Besides, the rate of UUI did not differ either. [52] As in our work, the number of studies included in the meta-analysis was limited. The missing data might prevent the identification of a difference between the various surgical techniques. However, the overall rate of long-term UI is low. In our systematic review, 16 studies presented UI data. Of those, 11 reported that no patient (EEP and TUR-P) suffered from UI 12 months after surgery. The highest UI rate reported was 4% [26].

In terms of surgical efficacy, there might be a disadvantage for HoLEP and a slight advantage for BipolEP. First, HoLEP seems to be the “slowest” surgical technique. On average, surgery in HoLEP took 15.78 min longer than in TUR-P, 16.72 min longer than in BipolEP, and 22.41 min longer than in DiLEP. The difference between HoLEP and BipolEP could partly be explained by the fact that all of the BipolEP studies used for the analysis, with the exception of Geavlete et al. [36], removed prostatic tissue without a morcellator. Because only in recent years, this procedure gained popularity for BipolEP as well [53]. However, since in all DiLEP studies, prostatic tissue was removed by morcellation too, it is unlikely that the process itself is the only cause for prolonged surgery. Second, the most prostatic tissue was removed in BipolEP. On average, an additional 13.65 g of prostatic tissue had been removed in BipolEP compared to TUR-P. However, pairwise indirect comparison between the enucleation methods (HoLEP, ThuLEP, DiLEP, and BipolEP) showed no statistically significant difference. It is worth mentioning that Funnel-Plot and Egger's test for asymmetry showed the suspicion of a possible publication bias for the endpoint resection weight. This is indeed plausible, as the resection weight is a complex parameter carrying the risk of misleading results. Its measurement is subject to many sources of error. For example, the weight varies depending on the time of measurement and thus on the degree of moisture. It is also altered by the proportion of tissue that has been vaporized and, therefore, depends on the energy source used. For instance, a greater proportion of tissue is getting vaporized when a laser, like the thulium laser, is being used compared to a resection loop with an electric current. As an alternative to resection weight, the postoperative drop in PSA value can be used to measure the extent of tissue removal. It is not biased by the resection/vaporization ratio and the time of measurement is of less importance. Of course, there are also potential confounders that can alter the PSA value, for example, the inflammation of prostatic tissue. Therefore, an accurate prediction on the extent of tissue removal can only be made if both parameters are taken together. However, the studies selected for this meta-analysis lack these pieces of information. The missing data should be considered a drawback.

Nevertheless, the surgery time and resection weight results show an advantage for BipolEP in terms of surgical efficacy—operation time is short, the amount of resected prostatic tissue is high.

Due to the more complete removal of the prostatic tissue, enucleation of the prostate leads to a better postoperative result than conventional TUR-P. Although this only becomes statistically significant 12 months after surgery. Direct comparison between HoLEP and TUR-P showed a significantly lower IPSS (− 0.89 points) 12 months after surgery for HoLEP. A pairwise indirect comparison between the enucleation methods (HoLEP, ThuLEP, DiLEP, and BipolEP) also showed a lower IPSS (− 1.4 points) twelve months after surgery for HoLEP in comparison with ThuLEP. However, whether an IPSS lower by one point is of clinical relevance remains to be questioned. Even if a one-point difference for an IPSS of four means an increase or decrease of 25%, it has little effect on the patient’s well-being. For this reason, comparing the Qmax in the uroflowmetry is much more relevant for assessing the success of the procedure. Twelve months after surgery, HoLEP showed a statistically relevant higher Qmax of 1.04 ml/sec. than TUR-P. The same applies to BipolEP, showing a greater Qmax of 1.1 ml/sec. than TUR-P. Pairwise indirect comparison between the enucleation methods (HoLEP, ThuLEP, DiLEP, BipolEP) also showed an advantage for HoLEP and BipolEP compared to DiLEP. Their Qmax was 1.94 ml/sec. and 2 ml/sec. higher than that of DiLEP. There was no difference between HoLEP, ThuLEP, and BipolEP. Even though an alteration of the Qmax of 1–2 ml/sec. might also not affect the patient's well-being, uroflowmetry still is a better surrogate-marker for the postoperative results than the IPSS alone. Furthermore, it is a predictor for the durability of relief from mechanical obstruction. Summarizing the functional outcomes 12 months after surgery, endoscopic enucleation, regardless of the source of energy in use, leads to better postoperative results than conventional TUR-P.

This meta-analysis clearly points out the advantages of endoscopic enucleation. It is an overall safe procedure, with a shorter postoperative catheter retention time and better functional results than conventionally performed TUR-P. The main endpoints among the various enucleation methods are similar, though the energy source (laser vs. electric current) impacts hemostasis and surgical effectiveness.

However, other essential aspects of clinical practice have not been covered by this meta-analysis—such as costs. Due to the lack of study data, direct cost comparison between all enucleation methods (HoLEP, ThuLEP, DiLEP, BipolEP) and TUR-P proves impossible. However, it can be assumed that BipolEP is the most cost-effective enucleation method, provided bipolar TUR-P is already offered in the clinic. BipolEP is the only enucleation method that can be performed using the same standard equipment as for bipolar TUR-P. This makes the acquisition of new expensive generators obsolete, as would be the case for a laser-based surgical method. Yet these costs make all the difference. Schiavina et al. recently published a cost comparison between TUR-P, HoLEP, and open prostatectomy. In this study, which did not consider the equipment's primary acquisition costs, no difference in total costs between TUR-P and HoLEP was found. Although the direct surgical costs of HoLEP were estimated to be higher than those of TUR-P, the difference was compensated by an on average shorter hospital stay. [54].

Another important aspect that has not been covered by this meta-analysis due to lack of data are the differences in learning curves. Although some studies deal with the enucleation technique’s learning process, only one study compares it among the various techniques. This study, published by Enikeev et al. in 2018, compares the learning progress of three surgeons, of whom each learns a different enucleation technique (HoLEP, ThuLEP, and MEP—monopolar enucleation of the prostate). They prospectively examined the first 30 surgeries. For the surgeons learning a laser-based enucleation technique, the steepest increase in resection weight/minute, which they defined as an indicator for surgical skills, occurred between the tenth and twentieth surgery and was higher than that of the surgeon learning MEP. Consequently, the authors conclude that aptitudes pertaining to the endoscopic enucleation of the prostate can be acquired within 30 procedures if supervised by an experienced surgeon, whereby laser-based enucleation is learned faster [55]. However, this study’s results have to be interpreted with caution, as the trial has potential sources of bias. Above all, the small study collective of only one surgeon per surgical technique. Rather than comparing the simplicity of learning a new surgical technique, this study contrasts the individual surgeons’ dexterity. Yet, they draw the same conclusion as other study groups proposing a minimum number of twenty to fifty surgeries to master endoscopic enucleation of the prostate [56, 57]. Results that have been validated with objective/robust quality criteria such as the pentafecta (complete enucleation and morcellation, within < 90 min, without any conversion to standard TUR-P, with an acceptable experience of stress and difficulty) [56]. Nevertheless, they most likely apply to all endoscopic enucleation methods, regardless of the energy source in use [58] [59]. Only for DiLEP, no study specifies a minimum number that is needed for learning the surgical technique. When discussing the learning curve of endoscopic enucleation, it should be further mentioned that BipolEP bears an additional major advantage over the other enucleation methods. Throughout the entire surgery, conversion to conventional TUR-P appears always feasible. This allows obtaining the surgical technique in a step-by-step approach without experiencing strong fluctuations in the postoperative results. This approach is confirmed in a study by Xiong et al., which analyzes the learning curve for BipolEP. The study shows that the number of conversions from BipolEP to TUR-P starts to reduce following the thirtieth intervention [58]. In other words, the number of interventions, which is generally considered the benchmark for mastering the procedure.

The main advantage of this study is the homogeneity of the patient collective. Only studies whose patients would also have been suitable for TUR-P were used for this meta-analysis. This facilitates the comparison between the enucleation methods and strengthens the significance of the results.

Nevertheless, this analysis also has certain drawbacks. Most importantly, the data available varies greatly among the various enucleation techniques. There is a significant discrepancy in the number and quality of trials available and the number of patients included. Also, the trials have different primary endpoints, and therefore not always all information of interest has been reported. The heterogeneity of the studies could have biased our analysis. We tried to overcome this problem using Higgins *I*^2^ and Cochran’s Q to assess the studies' heterogeneity and either chose a fixed-effects or random-effects model.

Finally, when writing about endoscopic enucleation, there is a general dilemma: there are different acronyms for the respective surgical techniques, which may result in essential studies not being found. Further, the surgical techniques published under the same acronym are not always identical. They might differ due to differences in equipment, laser settings, or even alterations of the surgical steps. This issue was pointed out in a recently published narrative review from Maruccia S et al. comparing techniques for endoscopic thulium laser surgery of the prostate (60). Most notably, 42% of all included studies were classified as discordant in terms of procedural description. The problem was most frequently found in ThuVEP and ThuLEP. All three ThuLEP studies from our meta-analysis have disconcordant procedural descriptions—to a different extent. This comes as a drawback to our study. However, due to the procedure’s complexity, variations of the surgical technique are common in EEP. A further effort has to be put into the standardization of EEP. Reviewing the literature for similarities and differences in surgical technique is an essential first step in ascertaining this goal.

## Conclusion

Endoscopic enucleation of the prostate has several advantages over conventional TUR-P: a shorter postoperative catheterization time and an improved functional outcome 12 months after surgery. The differences between the enucleation techniques (HoLEP, ThuLEP, DiLEP, and BipolEP) are of lower significance. BipolEP is probably the most efficient surgical method, whereas the laser-based enucleation methods stand out due to superior hemostatic properties.

## Abbreviations

AEEP: Anatomical endoscopic enucleation of the prostate
BipolEP: Bipolar enucleation of the prostate
BPE: Benign prostatic enlargement
BPO: Benign prostatic obstruction
CD: Clavien–Dindo Scale
CI: Confidence interval
DiLEP: Diode laser enucleation of the prostate
EAU: European Association of Urology
EEP: Endoscopic enucleation of the prostate
IIEF: International Index of Erectile Function
IPSS: International Prostate Symptom Score
HoLEP: Holmium laser enucleation of the prostate
LoE: Level of evidence
LUTS: Lower urinary tract symptoms
MEP: Monopolar enucleation of the prostate
MD: Mean deviation
Ml: Milliliters
ml/sec.: Milliliters per second
NOS: Newcastle–Ottawa Assessment Scale
OP: Open prostatectomy
OR: Odds ratio
PkEP: Plasmakinetic enucleation of the prostate
PSA: Prostate-specific antigen
PVR: Post-void residual urine
Qmax: Maximum flow rate in milliliters per second on uroflowmetry
QoL: Quality of life
sec.: Second
SUI: Stress urinary incontinence
ThuLEP: Thulium laser enucleation of the prostate
ThuVEP: Thulium laser vapo-enucleation
TUR-P: Transurethral resection of the prostate
UI: Urinary incontinence
UUI: Urge urinary incontinence
