# Correction to: Endoscopic enucleation of the prostate (EEP). The same but different—a systematic review

**DOI:** 10.1007/s00345-021-03791-6

**Published:** 2021-07-26

**Authors:** M. Pallauf, T. Kunit, C. Ramesmayer, S. Deininger, T. R. W. Herrmann, L. Lusuardi

**Affiliations:** 1 Department of Urology, University Hospital Salzburg, Paracelsus Medical University, Salzburg, Austria; 2 Department of Urology, Spital Thurgau AG, Frauenfeld, Switzerland

## Correction to: World Journal of Urology 10.1007/s00345-021-03705-6

Unfortunately, the surname of the co-author T. R. W. Herrmann was misspelled in the original publication of the article. It is correctly spelled as Herrmann and not Hermann.

The original article has been corrected.

